# Perceptual Modalities Guiding Bat Flight in a Native Habitat

**DOI:** 10.1038/srep27252

**Published:** 2016-06-06

**Authors:** Zhaodan Kong, Nathan Fuller, Shuai Wang, Kayhan Özcimder, Erin Gillam, Diane Theriault, Margrit Betke, John Baillieul

**Affiliations:** 1 Department of Mechanical and Aerospace Engineering, University of California, Davis, CA 95616, USA; 2 Center for Ecology and Conservation Biology, Boston University, Boston, MA 02215, USA; 3 Division of Systems Engineering, Boston University, Brookline, MA 02446, USA; 4 Department of Mechanical and Aerospace Engineering, Princeton University, Princeton, NJ 08544, USA; 5 Department of Biological Sciences, North Dakota State University, Fargo, ND 58108, USA; 6 Department of Computer Science, Boston, MA 02215, USA; 7 Department of Mechanical Engineering, Boston University, Boston, MA 02215, USA

## Abstract

Flying animals accomplish high-speed navigation through fields of obstacles using a suite of sensory modalities that blend spatial memory with input from vision, tactile sensing, and, in the case of most bats and some other animals, echolocation. Although a good deal of previous research has been focused on the role of individual modes of sensing in animal locomotion, our understanding of sensory integration and the interplay among modalities is still meager. To understand how bats integrate sensory input from echolocation, vision, and spatial memory, we conducted an experiment in which bats flying in their natural habitat were challenged over the course of several evening emergences with a novel obstacle placed in their flight path. Our analysis of reconstructed flight data suggests that vision, echolocation, and spatial memory together with the possible exercise of an ability in using predictive navigation are mutually reinforcing aspects of a composite perceptual system that guides flight. Together with the recent development in robotics, our paper points to the possible interpretation that while each stream of sensory information plays an important role in bat navigation, it is the emergent effects of combining modalities that enable bats to fly through complex spaces.

Navigation through cluttered environments is a fundamental challenge for animal locomotion^1^,^2^,^3^,^4^,^5^,^6^. Animal navigation requires fusion of spatial memory with information from multiple sensory channels, forming a coherent representation of the environment. Bats rely on this composite representation to find their way from roosts to foraging locations that may be some distance away. Central to their sensing, echolocation allows insectivorous bats to compute relative distances and directions to nearby objects^7^,^8^,^9^ and to make inferences about object shapes^10^, but there is increasing evidence that vision plays a role as well^11^. In concert, these sensory modalities allow bats to construct detailed images of the surrounding world in egocentric coordinates, and imprinted on spatial memory neurons, the images allow bats to encode and retrieve information in allocentric (or world) coordinates^12^,^13^. By fusing heterogeneous information coming from diverse channels, bats are able to fly near and around obstacles and to navigate significant distances^14^.

Previous research on the flight behavior of bats in cluttered environments has been mainly conducted in carefully constructed laboratory settings with small experimental arenas^8^,^12^,^13^,^15^,^16^,^17^. In such a setting, it has been found that, in complex environments, big brown bats (*Eptesicus fuscus*) point and shift their sonar beam to sequentially inspect closely spaced objects and change their call rate in accordance with their distance to targets^16^. Placed into an arena with novel obstacles, bats quickly acclimate and, within days, acquire smooth and stereotypical flight paths that are traversed with decreased call rates^2^. Despite the centrality of echolocation in the portfolio of perceptual modalities used by bats, there is increasing evidence that visual cues are more important for bats flying through natural habitats than previously recognized, suggesting integration of multiple sensory modalities during flight^17^. How bats use a combination of sensory cues in combination with spatial memory and predictive navigation, especially within their natural habitat, has been an open question.

In order to capture the behavior of free-flying bats in their natural habitat, we conducted a field experiment by observing flight patterns of a colony of *Myotis velifer* over seven days in an artificial cave habitat located near Johnson City, Texas (Fig. 1). On the first day of observation, we recorded the *M. velifer* flying along their normal flight path as they emerged from the roost. On the second day, an obstacle (a padded PVC pipe) was placed in the normal flight path of the bats. The pipe was kept at the same location for five consecutive days and then removed on the seventh and last day (see Supplementary Methods for details on the experimental setup and data collection). Technology innovations for tracking animals with multiple thermal cameras (see Methods) provide a means to extract accurate locations of a group of flying bats on a millisecond-by-millisecond basis^18^. We were also able to record syncronized echolocation calls along with the thermal videos (see Methods). The data together presents us a unique opportunity to understand how bats combine information from multiple sensory modalities as well as spatial memory to enable their agile and robust flight. Our main observations include: (i) the bats were able to adapt to the newly introduced obstacle and develop stereotypical flight patterns within days, demonstrating the development of spatial memory; (ii) some bats reacted to their neighbors before they received the echoes from those bats, indicating the possible role of other sensory modalities (e.g., vision or predictive navigation) to enable agile flight within cluttered spaces and in groups. To our knowledge, our paper is the first instance in which free ranging bat flight behavior around obstacles in their natural habitat has been recorded, detailed, and analyzed in the context of multiple sensory modalities.

## Results

### Temporal and spatial changes of bat flight behavior after the introduction of a novel obstacle

**Figure 2:**
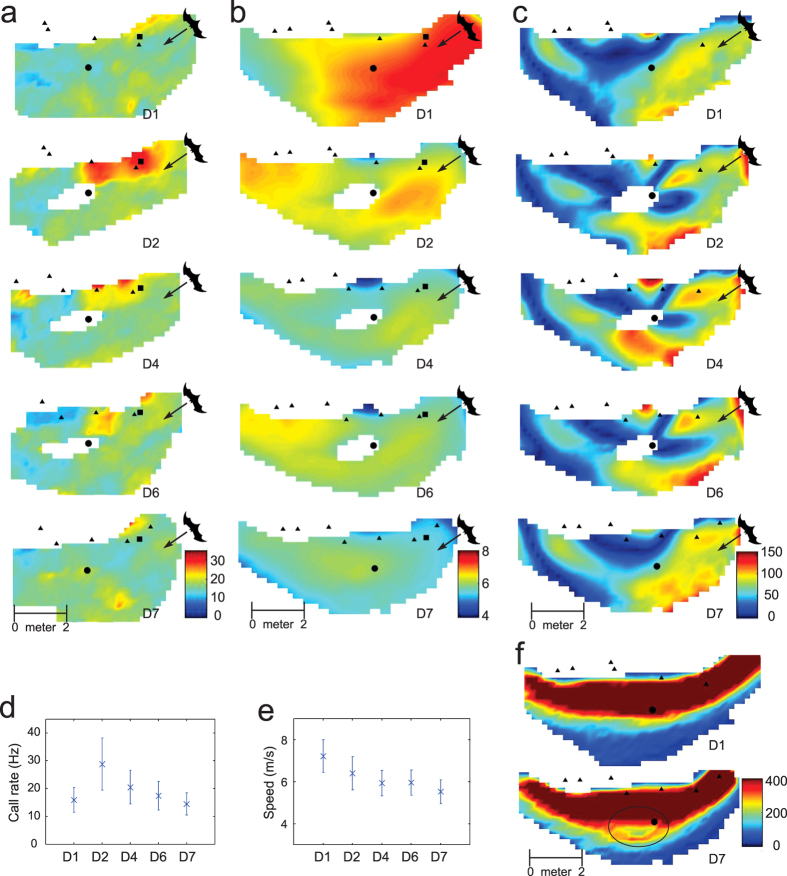
Histograms showing the effects of spatial memory on the flight behavior of bats. The histograms of (**a**) mean call rate (in Hz), (**b**) mean speed (in meter per second) and (**c**) mean turning rate (in radian per second) are shown for days 1, 2, 4, 6, and 7. Black triangles mark key feature locations (e.g., tree branches). Black dots mark locations of the obstacle (its virtual locations on days 1 and 7 were shown for comparison). The evolution of (**d**) call rate and (**e**) speed is shown for the positions marked as black squares in (**a**,**b**). (**f**) shows the occupancy histograms of day 1 and day 7. The black dots mark the virtual locations of the pole.

Because the environment remained essentially constant over the period of observation (except for minor weather variations), we pooled data from all five experimental days, and provide spatial histograms (see Methods and Fig. 2) and principal component analysis (see Methods and Fig. S6) to study both temporal changes (day-to-day) and spatial changes (altered flight paths) in the way that the bats moved. On the day that the PVC pipe was placed in the flight corridor (day 2), sharp maneuvers were observed as the bats approached the obstacle (Fig. 2c). The bats began their avoidance maneuvers progressively further from the obstacle on successive days – indicating increasing awareness of its presence. By using the concept of passing ray (see Methods), we are able to define the mean trajectory for a group of bats as well as the distribution of the group around the mean trajectory as shown in Fig. S5. All bats passed the obstacle from roughly the same direction, and the variance in individual trajectories with respect to the group mean trajectory decreased on successive days (Fig. S6), implying a return to stereotypical trajectories throughout the colony. In particular, the mean trajectory of day 6 stabilized to a level that was significantly different from that of day 2 (with p = 2.62 × 10^−6^ according to the Kruskal-Wallis ANOVA). From a large set (~10,000) of reconstructed trajectories, we selected 114 that were clearly associated with a single bat for the purpose of audio analysis (see Methods). Mean call rate on day 2 increased dramatically from that of day 1 (Fig. 2a) for a large region (2 meter by 1 meter) located in the direction from which the bats approached the pipe. Following day 2, both the area with high call rates as well as the mean call rate near the obstacle decreased as the bats became acclimated to the obstacle (Fig. 2a,d). By day 7, the mean call rate stabilized to a level that was not significantly different from that of day 1 (with p = 0.02 according to the Kruskal-Wallis ANOVA). The occupancy histograms (see Methods) of day 1 and day 7 are shown in Fig. 2f. It can be observed from the figure that a noticeable fraction of the bats (marked by the black oval) deviate from the baseline behavior in day 1, indicating a lingering memory of the obstacle.

### Possible role of vision and/or predictive navigation suggested by the heading delay analysis in leader-follower pairs

**Figure 3:**
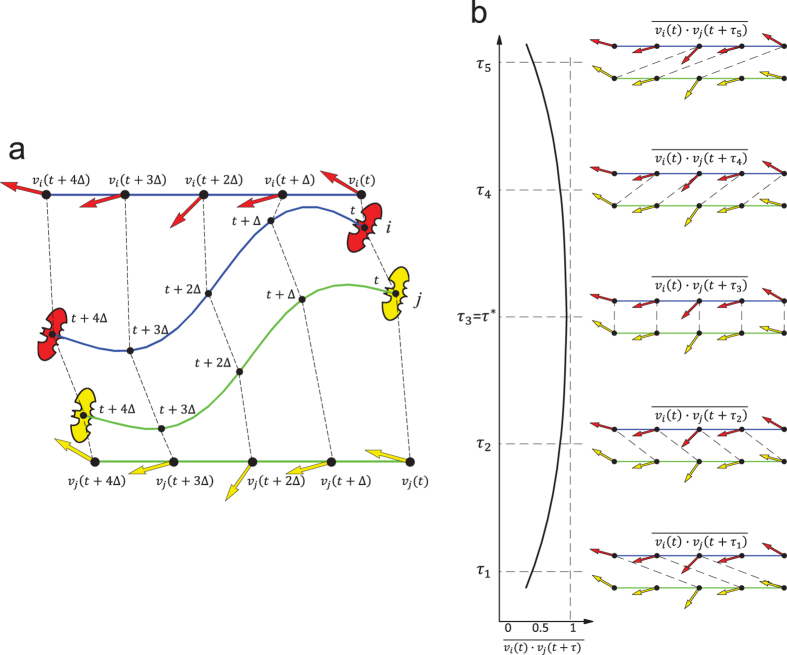
Summary of the heading delay definition for a leader-follower bat pair. (**a**) is a cartoon representation of a typical leader-follower pair with the red bat being the leader (bat *i*) and the yellow one being the follower (bat *j*). The arrows indicate the direction of motion at each time frame (with ∆ as the sampling time). For the bat pair (*i, j*), the heading correlation function is given by 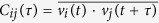. The heading delay (a concept similar to the directional correlation delay introduced by Nagy *et al*.^19^) of the bat pair is then defined as 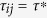 that corresponds to the maximum value of *C*_*ij*_(*τ*) as visualized in (**b**).

**Figure 4:**
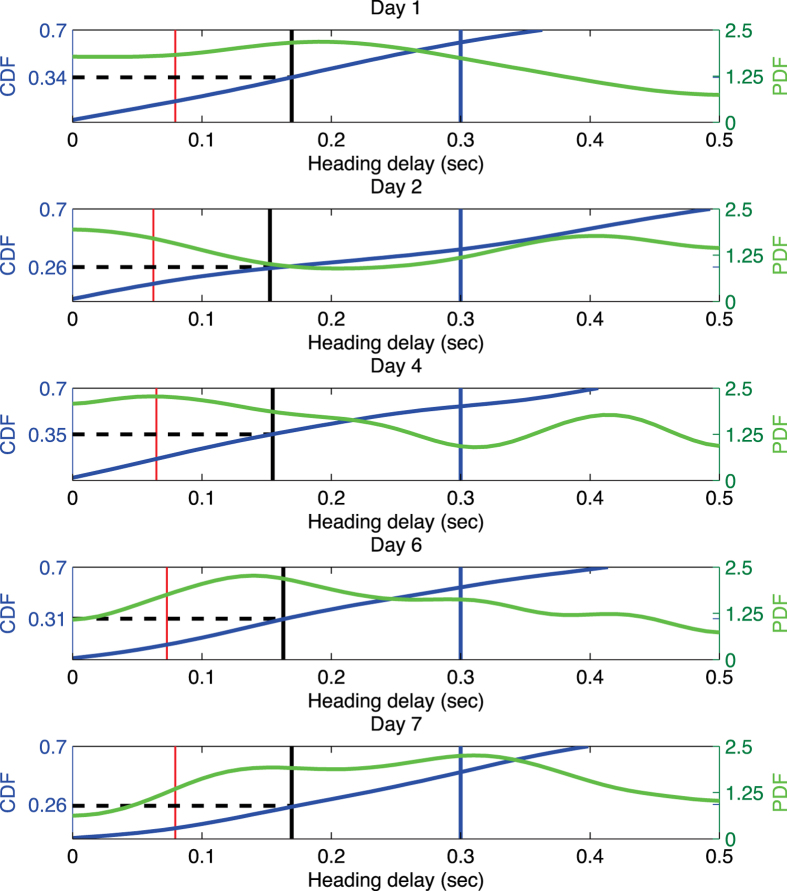
Heading alignment in leader-follower bat pairs is frequently too rapid to be explained by echoes returning from the followers’ vocalizations. Horizontal axes denote follower bat heading delay values, indicating the time required for the follower to align with the leader. The green curves and blue curves show the PDFs (probability distribution function) of heading delays, and the CDF (cumulative distribution function) of heading delays, respectively. Vertical lines mark significant temporal values: red designates the end of the first call interval; black is one call interval time plus the auditory reaction time^39^ (90 ms), and the blue line is the average reaction time of maneuvering bats in a previous study^23^ (300 ms).

This project also addressed flight behaviors of leader-follower pairs of *M. velifer.* Our goal was to study the spatial and temporal relationships of pairs of bats to understand how the follower was reacting to the leader, and what sensory modalities were being used by the follower. Heading delay analysis (see Methods and Fig. 3 for details) was used to identify leader-follower pairs^19^,^20^. It provides a measure of the time needed for a change in flight direction to propagate from a leader to a follower - specifically how long it takes for the follower to realign itself with the flight direction of the leader after the leader has turned. Among over ten thousand reconstructed trajectories recorded over a seven day period, we selected 277 pairs of trajectories (see Table S3 for details) in which there were two bats in the field of view throughout the flight corridor. Such bat pairs were considered to be a leader-follower pair if the following criteria were met: (a) the distance between the leader bat and the follower bat was less than 1 meter when they entered the field of view of all cameras; (b) there were more than 3 meters between the leader-follower pair and the next closest bat (we wanted to minimize the likelihood that the follower might be reacting to the activity of a bat other than the one we identified as the leader); (c) the maximum heading correlation (as described in Methods) between the leader bat and follower bat was greater than a threshold value of at least 0.6. The results for values between 0.6 and 0.8 are essentially the same and for the case 0.8 are as follows. We placed the heading delay values of the bat pairs into 20 bins of temporal width 50 ms and counted the fraction of pairs falling into each bin. These fractions were then smoothed to obtain a probability density function (PDF). Interestingly, more than 26% of all the observed leader-follower pairs had a heading delay that was less than one call interval time plus the minimum neuromuscular reaction time (Fig. 4). In other words, for this set of pairs, the time interval between the maneuvers of the leader and the follower is too short to be explained by echoes returning from the follower’s vocalizations. This pattern suggests that some followers may not be using echolocation as their primary sensory mechanism for aligning their flight with a leader. Further, the probability density functions described for days 2 and 4 in Fig. 4 are clearly bimodal, in contrast to the unimodal PDF of day 1, implying the possibility that not all follower bats are using the same perceptual cues to guide their flight on days when the artificial obstacle was in place.

## Discussion

Among over ten thousand bat trajectories that were studied to prepare this paper, we have noted the emergence of stable standard flight behaviors as the bats became increasingly familiar with the obstacle that had been placed in their normal flight path. Our observations, which illustrate the effects of spatial memory on flight and vocalization behaviors of bats, are in agreement with observations from laboratory experiments^2^,^12^. When a new obstacle is introduced, a bat increases its call rate in order to avoid colliding with the obstacle and to sample the new surroundings. The presence of the same obstacle at the same location on subsequent days reinforces spatial memory. Once able to anticipate the presence of the obstacle, the bat can turn to avoid it sooner. Once the obstacle has been registered into spatial memory, its flight pattern will be stabilized, and it can navigate around the obstacle with a normal call rate. After the removal of the obstacle (day 7), some bats behaved as if the obstacle was still in place as shown in Fig. 2f. Instead of flying in smooth and relatively straight trajectories as in day 1, these bats turned roughly at the original location of the obstacle. Such a behavior is a strong indication of utilization of spatial memory in free flying bats.

Due to the relatively lower acuity of insectivorous bat vision compared with echolocation, the main role of a bat vision has been assumed to be for long-distance use, where visual detection range exceeds echolocation range^21^. Only recently has evidence emerged to suggest that short-range visual capabilities are better developed than previously supposed and, at least under certain conditions, vision plays a significant role in navigating near clutter^11^,^17^. In carefully designed laboratory experiments, Holler and Schmidt^22^ have demonstrated that bats may ignore echoacoustic information in cases where it contradicts visual information. They note, however, that there was some uncertainty in their conclusions due to the artificial setting in which the behaviors were observed. It is thus of interest that the Holler and Schmidt findings are consistent with our field observations of bat pairs where the follower frequently reacted to changes in the heading of a leader more quickly than would be possible if the follower was relying on echoacoustical information alone.

For bat pairs, the rapid rate with which the follower bats align themselves with leaders suggests perceptual modalities other than echolocation are being employed in navigation. Among the possibilities, it is natural to speculate that as the bats acclimate to the unexpected obstacle, they will naturally converge on similar pathways—regardless of whether they are traveling within a certain distance of each other or at the same time, which would apparently allow the followers to align with the leaders more quickly than would be the case if they were solely reacting to leaders’ movements. We discount this possibility, however, because if it were true, it would be expected that the correlation between the heading delay and the average distance between the bat pairs would be significant. In fact the opposite is true—as illustrated in the scatter plot of Fig. S10. The correlation is small on day 2 (0.34), and although the correlation became bigger on day 4 (0.40) and day 6 (0.51) as the bats acclimated to the environment, there was never a high correlation between distances between leaders and followers and the heading delays. Since the distance between the leader and follower in each of our bat pairs is fairly small, there is also the possibility that the follower is listening to the vocalizations or wing beats of the leader. Although the study described by Giuggioli *et al*.^23^ discounts eavesdropping as a sensory guide to navigation in settings like the one we observed, we cannot rule it out as playing a role.

An interesting bi-model pattern can be observed in the probability density functions (PDFs) of the heading delays of days 2, 4, and 6 as shown in Fig. S7. We draw a vertical line in each PDF, which is the mid-point between the highest and second highest peaks, for each day. We call those bat pairs falling to the left of the vertical line left mode pairs (indicated by a light green background in Fig. S7) and those falling to the right of the line right mode pairs (indicated by a light blue background in Fig. S7). Specifically, a bat pair belonging to the left mode of the distribution implies a rapid alignment between the leader and follower, and a pair belonging the right mode has a slower alignment between leader and follower. Among these two sets of bat pairs, it is of interest to note that there are some differences in the paths that were chosen to avoid the obstacle in the flight corridor. Some of the bat pairs pass the obstacle on the same side, while others split and pass the obstacle on opposite sides. Some examples are shown in Fig. S8. Note that Fig. S9 shows the percentages of each category for all bat pairs, those in the left mode of the PDF (rapid alignment) and those in the right mode (slow alignment). The result shows that the bat pairs in the left mode are primarily pairs with leader and follower flying on the same side of the pole, and the pairs in the right mode have a much higher chance of splitting. This implies the possibility that the bat pairs in the left mode are using some faster sensory modalities (e.g., vision) which enable the followers to robustly track the leaders, while the bat pairs in the right mode are using some slower sensory modalities (e.g. vocalization) so that the followers are more likely to miss the moment to pass the pole on the same side as the leaders. The possible reason for the different sensory modality choices might result from the different individual value assigned to sensory speed (vision > vocalization) vs. sensory precision (vision < vocalization). Further, there is a dramatic decrease of leaders and followers passing the obstacle on opposite sides from day 2 to day 4. This suggests the emergence of stable standard flight behaviors as the bats became familiar with the obstacle.

Vision may be only part of the suite of sensory systems in operation. For instance, it has been shown that bats use a predictive strategy to intercept prey^24^. The same mechanism may be adopted by the bats to track other bats. But further experiments and analysis are needed to settle the issue. It is worth pointing out that recent research has shown how the optical flow that is registered on the visual cortex can provide the steering cues needed to fly near and through clutter^25^,^26^, and to align a follower with a leader^27^. Consistent with previous work^23^, our analysis argues that vision plays an important role – in combination with echolocation, spatial memory, and possibly predictive navigation – in guiding the flight behaviors that were recorded in the field.

## Methods

### Ethics statement

The protocols used in this study were carried out in accordance with the American Society of Mammalogists guidelines (Sikes *et al*.^28^), and were approved by Boston University’s Animal Care and Use Committee (Protocol #11-021).

### 3D trajectory reconstruction with multiple thermal cameras

Our image processing strategy was similar to that employed in Betke *et al*.^29^. Bats were imaged against vegetation and clouded sky. The background was modeled using a running average with exponential decay. Foreground regions were identified using background subtraction with empirically determined thresholds. Multiple detected objects per foreground region were identified using brightness peaks, defined as local maxima that were sufficiently bright (thresholds determined heuristically) and sufficiently far away from other local maxima (based on apparent target size).

The cameras were spatially calibrated following the strategy used by Theriault *et al*.^18^, using the bundle adjustment algorithm. We followed the reconstruction-tracking approach described by Wu *et al*.^30^; image detections from each of the three cameras were used to reconstruct three dimensional object detections, using epipolar geometry and the DLT algorithm^31^. Although standard epipolar geometry requires only two camera views to determine 3D locations of points in the image, three cameras were used to deal with occurrences of bats being occluded by environmental clutter or by other bats. Redundant camera views were also found to be helpful in reducing noise and uncertainty. Tracks were constructed using multiple hypothesis tracking with a sliding window.

### Trajectory smoothing

Errors inevitably appear in these reconstructions due to the very small size of the bat images within the field of view and to uncertainties arising from occasional occlusions. Cubic splines were used to smooth the reconstructed noisy trajectories. Suppose a trajectory has *N* data points (*t*_*i*_, *p*_*i*_), where *p*_*i*_ is the *i*-th reconstructed 3D position and *t*_*i*_ is the corresponding time. A typical smoothing technique then assumes that the observed data result from the combination of a model *f*(*t*) and a Gaussian noise *ε* ~ *N*(0, *σ*^2^), i.e.,


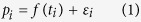


Our smoothing has assumed a cubic spline model for *f*. Dey and Krishnaprasad^32^,^33^ treat a somewhat more general 3-rd order generative model, and exploit group symmetries on SE(2) as an approach to data smoothing. In the case of the cubic spline smoothing method, *f*(*t*) is a cubic spline curve and the curve is obtained by minimizing the following cost


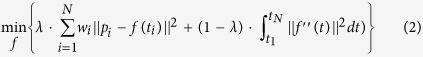


where *w*_*i*_ is the weight of the *i*-th data point *p*_*i*_, whose meaning will be explained below, the first term is the residual sum-of-squares, and the second term is a measure to penalize the roughness of the fitted curve. The smoothing parameter *λ* controls the tradeoff between the goodness-of-fit to the data and the smoothness of the curve. The term *f* ″(*t*) is the second derivative of the curve calculated at time *t*.

We assume that the precision of the reconstructed 3D positions is affected by the distance between the positions and the cameras, i.e., the further a position is away from the cameras, the less the precision of the reconstruction is. We used weighting to take this effect into consideration. Specifically the weight *w*_*i*_ of reconstructed position *p*_*i*_was set to be proportional to the reciprocal of the sum of squares of the distances between the position *p*_*i*_and the three cameras, i.e.,


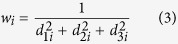


where *d*_1*i*_, *d*_2*i*_, and *d*_3*i*_ are the distances between the position *p*_*i*_ and the three cameras.

The solutions to the above reconstruction problem are sensitive to the smoothing parameter *λ*. Instead of specifying the parameter *λ* manually, we would like to choose a value λ^*^ that minimizes the true mean square error averaged over the weighted data points. The true mean square error is defined as *R*(*λ*) given by


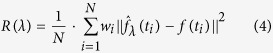


where 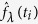 is the reconstructed trajectory when the smoothing parameter is set to be *λ*. Unfortunately, it is not practical to take the minimizer of *E*[*R*(*λ*)] as the optimum *λ* since *f*(*t*) is unknown. Fortunately, it has been demonstrated that, for measurements with high sampling rate, the generalized cross validation method^34^,^35^ is able to provide a good estimate of the minimizer of *E*[*R*(*λ*)]. The idea of generalized cross validation is that the best model for the measurements is the one that best predicts each measurement as a function of others. It performs multiple rounds of analysis of the data set. Each round involves partitioning a set of data into two subsets, the training set and the validation set, after which we perform the reconstruction on the training set, and we validate the reconstruction on the validation set. For a given value of *λ*, the mean square error is used to validate the fit in each round. The mean square errors are then averaged over all rounds associated with a given value of *λ*. The minimizer of the averaged validation errors is taken as the optimum λ^*^. We refer to Craven and Wahba^34^ and Golub *et al*.^35^ for more detail on generalized cross validation.

Based on the generalized cross-validation method to find the optimal parameter λ^*^ for each trajectory, Table S2 shows some statistical characteristics of the optimal smoothing parameter λ^*^ obtained by using generalized cross validation. We note that these statistics are fairly consistent from one day to the next. Figure S2 shows projected 2D trajectories of day 2 together with the locations of hot-pads and acoustic sensors.

### Smoothed trajectories in 2 and 3 dimensions

The portion of the bats flight corridor that was observed and recorded is approximately seven (7) meters in length and four (4) meters wide. The bats flew through this corridor at height ranging from roughly one to three (1–3) meters above the ground, with a typical animal entering the field of view at the high end of the range and descending to the lower end as it left the field of view. Smoothed flight paths in 3 dimensions were represented by cubic splines as described above. The raw three-dimensional point data were also projected onto a 2-dimensional plane that is parallel to the ground of the flight corridor. Smoothed trajectories in this plane were constructed using the same spline techniques. The animal speeds in both the two and three dimensional renderings turned out to be similar. The mean speed histograms in Fig. 2b accurately reflect flight speeds recovered from the 3-dimensional data.

### Passing ray

To quantify the convergence to a standard flight pattern, we formally compare trajectory segments up to the point at which they pass the pole obstacle. For a set of trajectories projected onto the plane, we define the associated passing ray as a ray (half line) originating from the pole and having a direction that is perpendicular to the average normalized velocity of the set. To carry this out, we first define


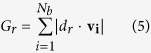


where *d*_*r*_ is the unit vector representing the direction of a ray, **v**_**i**_is the normalized velocity of *i*-th bat, · denotes a dot product operation, and *N*_*b*_ is the number of bats within the group. Then the passing ray is the ray that corresponds to a direction 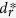 where *G*_*r*_ reaches its minimum, 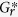. (See Fig. S4.) In an ideal case, the ray could be drawn such that each bat trajectory velocity vector is perpendicular at crossing; this would give 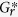 the value zero. While this is not the case with the flight data we have recorded, it is nevertheless the case that all velocity vectors make a steep angle in crossing the ray.

### Mean trajectory and principal component analysis

The position where a bat passes the pole, called the passing position, is then defined as the intersection between the bat’s trajectory and the passing ray (as described above). We shall parameterize the bat trajectories by arc length, with the zero value corresponding to the crossing point and the positive direction being opposite to the direction of flight. The position of a bat *i* along its path is given by the arc length variable *s*_*i*_ with *s*_*i*_ decreasing as the bat approaches and then passes the pole. As shown in Fig. S5, the mean trajectory of a group of bats can then be created by averaging the positions of all bats corresponding to the same arc length *s*. The variance of the distribution of bats at each *s* can be characterized by an ellipse. For all bat positions corresponding to a given value of *s*, a 2-by-2 covariance matrix can be generated. The 2-by-2 covariance matrix has two eigenvalues *λ*_min_ and *λ*_max_ (*λ*_min_ < *λ*_max_) with their corresponding eigenvectors *v*_min_ and *v*_max_, which, according to principal component analysis, represent the directions with the maximum and minimum variance, respectively. The lengths of distribution ellipse axes can then be calculated by taking the square root of the eigenvalues of the covariance matrix.

### Single bat selection

A bat was considered to be a single bat if the following criteria were met: (1) its trajectory was in the field of view of all cameras for more than 1 second (roughly half the length of the twenty meter flight corridor on average); (2) the distance between the bat and its closest neighboring bat was always more than 3 meters.

### Acoustic data handling

Among approximately 10,000 smoothed trajectories recorded over a five day period, we identified 114 single bats for audio analysis based on above criterion. The acoustic recording of each selected bat was first filtered through a high-pass filter with 5 kHz cut-off. A spectrogram such as the one shown in Fig. S3 was then computed by using a 1024 point fast Fourier transform^36^ (with 98.43% overlap). For the 250 kHz sampling rate used during the recording, this gave a 244 Hz frequency resolution. For each bat call, peak time was calculated from its spectrogram. The peak time was defined as the time corresponding to the maximal value of the power spectrum of the call. For two adjacent bat calls, the call interval time was defined as the time difference between their peak times. The call rate was then defined as the reciprocal of the time interval between two calls.

### Video and acoustic data synchronization

Shortly after the start of each video recording session, an electronic lighter was ignited about 30 cm away from the acoustic sensor arrays. An ignition produced a click sound and a flame at approximately the same time (the time difference between the click sound and the flame was assumed to be negligible). The click sound was captured by the acoustic sensors and the flame was captured by the thermal cameras. The click sound had a very short time duration and thus can be roughly viewed as a delta function in the time domain. The frequency range of a delta function in the frequency domain or in the spectrogram was quite different from that of a typical bat call. Thus, the time of a click could be easily identified from the spectrogram. The temperature of the flame was significantly higher than that of the trees and other objects in the background and thus would result in a bright spot in the thermal video which enabled us to locate the time of the ignition.

Once the times corresponding to the ignition were identified in both the video and the audio recordings separately, they were used as references to synchronize the video and audio data. After synchronization, relationships between bat vocalizations and flight dynamics could be studied. For instance, call rates could be correlated with flight speed, flight path curvature, and so forth.

### Spatial histograms and occupancy histograms

In order to visualize and quantify how bats react to spatially distributed environmental features, such as the novel obstacle (PVC pole introduced on the second day of the experiment), the concept of “spatial histogram”^37^ is used to visualize the synchronized dataset. The spatial histogram can be understood as follows. For each 2D location *p* = (*x, y*), there exists some variable *o*(*p*) of interest – speed, turning rate or call rate in our study. The spatial histogram *f*(*o*) contains occurrence information of the variable of interest at the given location (*x, y*). In the paper, the 2D dataset of bats, which can be seen as consisting of samples of the variable *o*, will be used to approximate *f*(*o*), in particular its mean and variance within cells in a grid.

The 7 meter by 4 meter flight corridor was partitioned into a grid of 0.2 meter by 0.2 meter rectangles. We then represent *f*(*o*(*p*)) in each grid cell by its sample mean


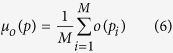


and its sample variance


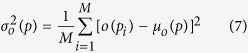


where 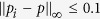 meter, i.e., the position *p*_*i*_ should fall into a square that is centered at position *p, M* is the total number of all the data points falling within the square. To visualize the number of data points falling within each square, we set *f*(*o*(*p*)) = *M* to generate the occupancy histograms (a concept introduced by Barchi *et al*.^2^).

One concern regarding the choice of grid size is the need to balance the tradeoff between bias and variance of the estimates, similar to any averaging or smoothing process. Generally speaking, a larger cell size results in a smoother estimate but at the same time may introduce a larger bias. The technique introduced in Mettler and Kong^38^ was used to determine the grid size (0.2 meter in our case). The key is to make sure the chosen grid size is consistent with the data sparsity as well as the spatial spectrum of bat flight behavior. Figure 2a–c show the histograms of mean velocities, turning rates and call rates, respectively.

### Heading delay

A *following event* is said to happen in a bat pair when a bat’s sequence of motion directions is “copied” by another bat with a certain delay. We define the heading delay (a concept similar to the directional correlation delay introduced by Nagy *et al*.^19^) to quantify such an event. For a bat pair (*i, j*), consisting of a leader *i* and a follower *j*, the heading delay *τ*_*ij*_ is defined as


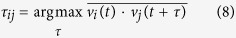


where *v*_*i*_(*t*) is the normalized velocity of the *i*-th bat at time *t*, ‘·’ denotes a dot product operation, and the overbar denotes a time average operation. The heading delay measures the time delay needed to shift the velocity *v*_*j*_(*t*) with respect to *v*_*i*_(*t*) to maximize their agreement.

Table S3 shows the number of bat pairs that have correlation larger than different thresholds. The threshold is set at 0.8 for Fig. 4.

## Additional Information

**How to cite this article**: Kong, Z. *et al*. Perceptual Modalities Guiding Bat Flight in a Native Habitat. *Sci. Rep.* **6**, 27252; doi: 10.1038/srep27252 (2016).

## Supplementary Material

Supplementary Information

## Figures and Tables

**Figure 1:**
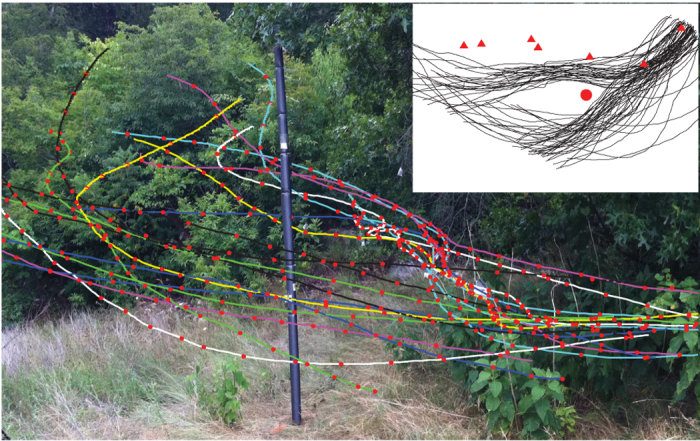
Audio and video data are synchronized and then overlaid with the environment to study the variations of bat behavior spatially and temporally. The flight corridor is about 7 meters long and 4 meters wide with *Myotis velifer* flying from the right (where their cave roost is located) to the left (towards their foraging area). A PVC pipe was placed in the middle of the flight corridor from day 2 to day 6. Three dimensional flight trajectories (colored curves are sample tracks from day 2) were then synchronized with bat calls (their locations are shown as red dots) during post-processing of data. –Photograph background by Z. Kong; three dimensional trajectories and overall image composition by S. Wang. The upper-right inset shows some reconstructed trajectories on day 2 where red triangles indicate the locations of hot-pads and red circle indicates the location of the hot-pad attached to the pole.
