# Supplementary material for: Perceptual Modalities Guiding Bat Flight in a Native Habitat
Source: Sci Rep. 2016 Jun 6;6:27252. doi: 10.1038/srep27252 (PMC4893665; doi:10.1038/srep27252)
Supplement: Supplementary Information [file srep27252-s1.doc]

**Supplementary Information**

**Perceptual Modalities Guiding Bat Flight in a Native Habitat**

**Zhaodan Kong^1^, Nathan Fuller^2^, Shuai Wang^3^, Kayhan Ozcimder^4^, Erin Gillam^5^, Diane Theriault^6^, Margrit Betke^6^, and John Baillieul^3,7,*^**

Department of Mechanical and Aerospace Engineering, University of California, Davis, CA 95616, USA

^2^Center for Ecology and Conservation Biology, Boston University, Boston, MA 02215, USA

^3^Division of Systems Engineering, Boston University, Brookline, MA 02446, USA

^4^Department of Mechanical and Aerospace Engineering, Princeton University, Princeton, NJ 08544, USA

^5^Department of Biological Sciences, North Dakota State University, Fargo, ND 58108, USA

^6^Department of Computer Science, Boston, MA 02215, USA

^7^Department of Mechanical Engineering, Boston University, Boston, MA 02215, USA

^*^Corresponding author. Department of Mechanical Engineering, Boston University, 110 Cummington Street, Boston, MA 02215. Email: [johnb@bu.edu](mailto:johnb@bu.edu)

Supplementary Methods

**Experimental Setup.** Fight behaviors of members of a *Myotis velifer* colony emerging from a cave roost were recorded using high-speed, high-resolution thermal video together with a bioacoustic recording system. All recordings were made at a single site in central Texas. The Bamberger Ranch Preserve is a 5500 acre wildlife and conservation preserve located near Johnson City, Texas and is home to an artificial roost structure that was designed to attract and house bats. Habitat surrounding the artificial cave is predominantly temperate savanna and juniper forest. A number of water sources are located within a kilometer of the roost, including vernal pools, flowing streams, and artificial lakes. Surrounding land usage is predominately livestock grazing.

The man-made cave houses two species of bat — Brazilian free-tailed bats (*Tadarida brasiliensis*) and cave myotis (*M. velifer*). While the two species cohabit, they do not roost in the same locations within the cave. Population estimates suggest approximately 250,000 Brazilian free-tailed bats and several thousand cave myotis use this roost. Upon emergence, cave myotis take two paths away from the cave. One path is a straight flight out of the mouth of the cave toward the service road. The bats will follow this route to nearby water sources. Another path taken by the bats involves a 180 degree left turn after leaving the cave after which the animals fly over the top of the cave following a tree line along a low grass field. Bats flying this path travel along a slowly rising hill that peaks and falls within 250 m of the cave entrance. Our thermal camera array was placed at the peak of this short hill, facing a portion of the flight path where overhanging trees force a natural bottleneck where bats are forced to navigate a complex environment within a very short distance and without foresight (their view is blocked by the hill). Based on visual observation, there appeared to be roughly equal numbers of bats traversing each path.

Raw flight data on *Myotis velifer* were collected shortly after sunset (approximately 20:30 CST) on July 13, 14, 16, 18, and 19, 2013. (In this document as well as the main text, we will call July 13 as Day 1, July 14 as Day 2 and so forth.) The experimental setup and weather condition (based on human observation in conjunction with historical weather record of Johnson City, TX obtained from http://www.wunderground.com) of each day are shown in **Table S1**. A pole, which was chosen as the obstacle the bats had to navigate around, was a PVC pipe wrapped with black flexible elastomeric foam to protect bats in case of collision. The diameter of the pipe with the form was about 8 centimeters and its length was about 2.5 meters. The pole was stuck firmly to the ground and placed about 1 meter away from the overhanging tree branches (as shown in the upper right image of **Fig. S1**). Its location was chosen by our team in such a way that it approximately split the bat column into two halves. Further, the upper tip of the pole was pushed into the canopy of the trees so that it was impossible for the bats to fly over the pole. **Table S1** also shows the availability of video and audio data. Audio data was unavailable for day 5 because there was a thunderstorm shortly before the bats’ emergence time, and acoustic sensors were not deployed to avoid probable damage. Video data was unavailable for day 3 due to the loss of the calibration data of that day.

**Thermal Video Collection, Extraction, and Storage.** Data were collected using 3 FLIR ThermoVision SC8000 thermal cameras and 3 FLIR/IO Industries High Speed Data Recorders (HSDR) running ThermaCAM® RTools™ v2.4. Cameras were placed following [1] to optimize 3D reconstruction accuracy. The cameras were placed in a line approximately perpendicular to the flight direction of the bats. Camera viewing angles (as shown in the lower right image of **Fig. S1**) were selected so as to optimize reconstruction accuracy at points of direct interaction between bats and the pole, and to maximize flight track duration.

Videos were collected at maximum resolution (1024×1024) and maximum frame rate (131.5 Hz) with a 5 ms integration time. Each camera was independently corrected with a built in non-uniformity correction to provide clear images free of optical artifacts, bad sensor pixels, and self-detection. Cameras were hardware-synced using GW INSTEK SFG-1013 function generator. Time stamps were synced to one computer clock. Recording was triggered by a remote start cable. Efforts were taken to minimize illumination of the flight corridor by computer screens and to minimize noise from computer cooling fans.

Video recording began shortly after sunset each night (approximately 20:30 CST). About half an hour before each night’s recording, nine hot-pads were attached to either the bushes or tree branches. These hot-pads provided reference points for later data analysis. They roughly defined the boundary of the bats’ flight path. However, two hot-pads were attached to bushes where the bats could fly over (marked as triangle A and B in **Fig. S2**). An effort was made to attach the hot-pads at the same locations each day. During the nights when the pole was present, two additional hot-pads were attached to the pole.

Start time was determined by researchers who were watching the thermal feeds. When a bat was detected in one of the feeds, a signal was given to begin recording. Each recording was 10 to 15 minutes long and collection was stopped after 30 minutes. The team took this approach of making short clips of data to limit file size, as these cameras output extremely large files (approximately 160 GB per 5 minutes of video at max frame rate and resolution), and to limit losses if a file had been corrupted during recording. Once recording ended, the team disassembled the camera array and reassembled it in the field headquarters to extract the videos. Using built-in software ThermaCAM® RTools™ v2.4, videos were extracted the night of recording and stored on 2 TB hard drives (Western Digital Caviar Green). Extraction was conducted each night because the process takes 10-12 times longer than the length of the actual recording, thus attempting to extract more than one hour of video per evening would have been time prohibitive. Data drives were stored in static-free bags and hard drive shipping containers while in the field.

**Acoustic Data Collection.** The bioacoustics recording system consisted of six condenser microphones (Avisoft CM16; frequency response ±5 dB between 10 and 100 kHz; Avisoft Bioacoustics, Germany) mounted in two arrays. They were placed near bushes (as shown on the left image of **Fig. S1**) to keep their effects on the bats minimal. Each array consisted of a central microphone mounted 1.3 m from the ground and 1-3 arms, each with a microphone mounted at the distal end (arm length = 1.1 m). Arrays faced towards the PVC pole and microphones were oriented on the horizontal plane. Array components were padded with pipe insulation and foam to minimize injuries to bats striking the setup. During a recording period, high-speed data acquisition was carried out using two Avisoft UltrasoundGate 416 units, each attached to a laptop running Avisoft Recorder. Recorded files were 5 min in length, with no temporal gap between files.

Supplementary Tables

| Day | Pole presence | Availability of video | Availability of audio | Weather condition |
| --- | --- | --- | --- | --- |
| 1 | no | yes | yes | scattered clouds |
| 2 | yes | yes | yes | mostly cloudy |
| 3 | yes | no | yes | rain |
| 4 | yes | yes | yes | scattered clouds |
| 5 | yes | yes | no | scattered clouds |
| 6 | yes | yes | yes | overcast |
| 7 | no | yes | yes | clear |

**Table S1. Experimental setup, availability of video and audio data and weather conditions.**

|  | Day 1 | Day 2 | Day 4 | Day 6 | Day 7 |
| --- | --- | --- | --- | --- | --- |
| $\mu(\lambda^{*})$ | 0.8112 | 0.8160 | 0.8286 | 0.7886 | 0.7688 |
| $\sigma^{2}(\lambda^{*})$ | 0.0429 | 0.0467 | 0.0424 | 0.0511 | 0.0514 |

**Table S2. Means and variances of optimal smoothing parameter.**

| Threshold | Day 1 | Day 2 | Day 4 | Day 6 | Day 7 | Sum |
| --- | --- | --- | --- | --- | --- | --- |
| 0.6 | 64 | 55 | 48 | 51 | 72 | 290 |
| 0.7 | 63 | 53 | 47 | 49 | 70 | 282 |
| 0.8 | 61 | 52 | 47 | 47 | 70 | 277 |

**Table S3. Number of bat pairs having correlation larger than different thresholds.**

Supplementary Figures

**Figure S1. Experimental arena.** The portion of the flight corridor where our team made video and audio recordings is about 7 meters long and 4 meters wide with bats flying from right towards left. A PVC pole (shown in the upper right image) was placed in the middle of the flight path. Three high-speed thermal cameras (shown in the lower right image) and six ultrasound-sensitive microphones (shown in the left image) were placed in such a way that they were pointing towards a direction that is approximately perpendicular to the flight direction of the bats while slightly towards the pole. –Site photographs and composite image by Z. Kong.


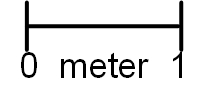
**
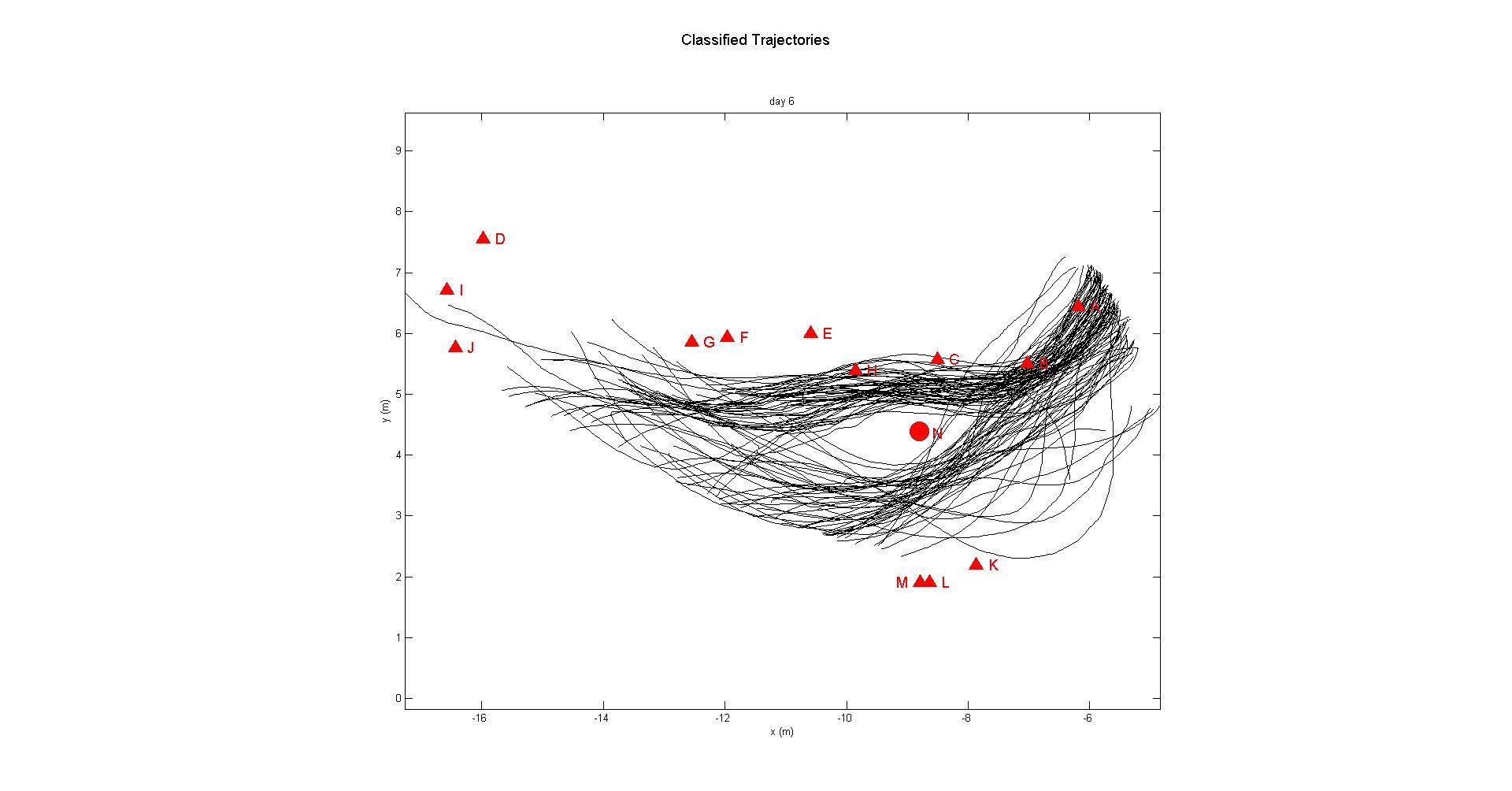
**

**Figure S2. Selected smoothed bat trajectories of day 4.** Red triangles indicate the locations of hot-pads (A-J) and acoustic sensors (K-M). Bats flew from the right towards the left. Hot-pads A and B were attached to low-growing bushes thus bats could fly over them. Red circle indicates the location of hot-pad N which was attached to the pole.

**Figure S3. Typical spectrogram of a single vocalization of *M. velifer*.**

a

b


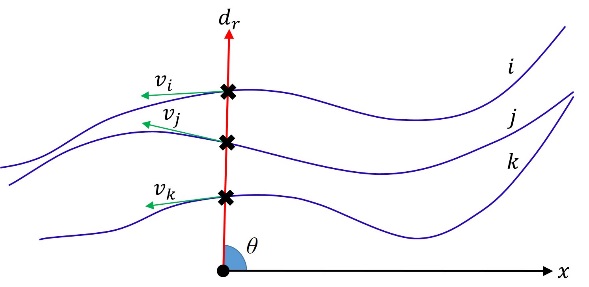


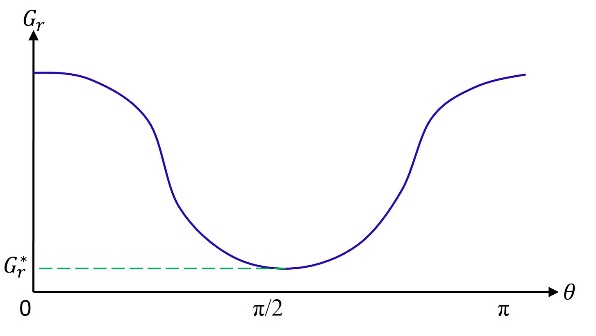


**Figure S4. Illustration of the concept of passing ray.** The blue curves in (**a**) represent the trajectories of a group of bats; the black dot indicates the location of the pole; the red arrow indicates a ray the direction of which is *θ* with respect to the *x* axis (black arrow) of the world coordinate; the black crosses mark the position where the ray intersect the trajectories; the green arrows indicate the bat velocities at the intersecting positions. *G_r_* is then the summation of the absolute values of the dot product between the unit vector representing the direction of the ray and the normalized velocity of each bat. (**b**) shows how *G_r_* changes with the direction of the ray.

a b


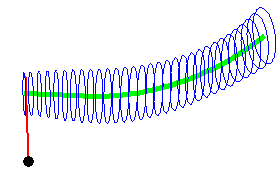

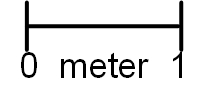

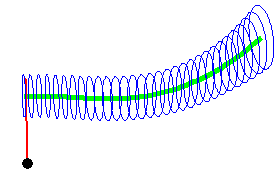

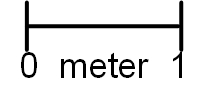


**Figure S5. Group mean trajectories.** (**a**) and (**b**) show the mean trajectories (green) and the distribution ellipses (blue) of day 2 and day 6, respectively. The black dots indicate the position of the pole and the red lines indicate the passing rays.

**Figure S6.** **Decrease in the variances of bat groups with respect to their mean trajectories is supporting evidence for the emergence of stereotypical trajectories due to spatial memory.** The colored curves show the changes of length of the major axes of the distribution ellipses (some examples are shown in **Fig. S6**) with respect to the arc length distance from the passing ray of the pole, *s*, on each day. Only those bats passing the pole from the right side on days 2, 4 and 6 are considered. For comparison, the same statistics of day 1 and day 7 are also shown (a virtual pole is used to classify bat trajectories).


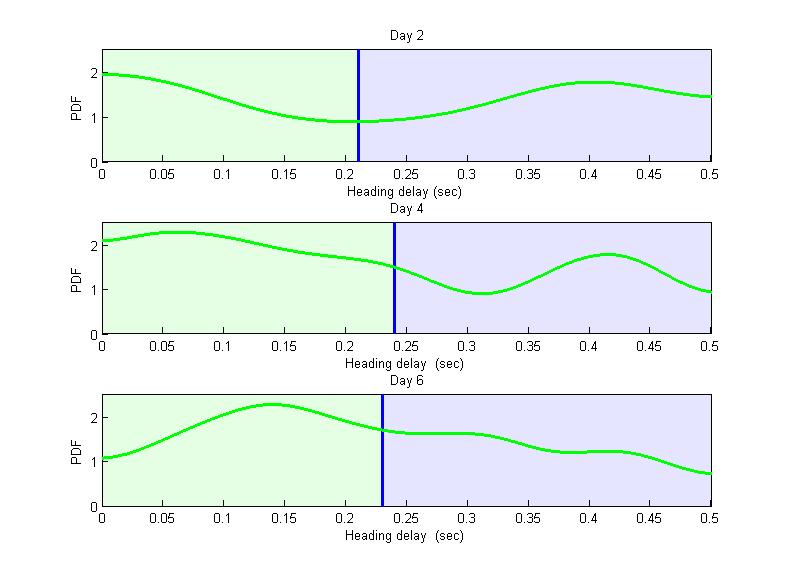


**Figure S7.** **Bi-modal pattern.** The probability density functions (PDFs) of heading delays are shown as green curves on days 2, 4, and 6, respectively. The same curves are shown in **Fig. 4** of the main text. The blue vertical lines are chosen as the mid-point between the highest and second highest peaks.

a b c


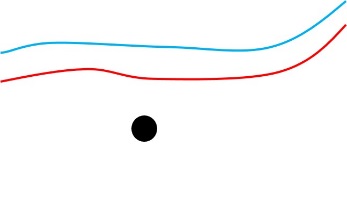

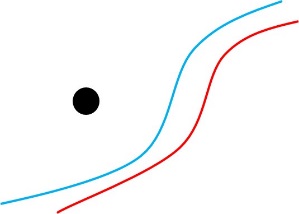

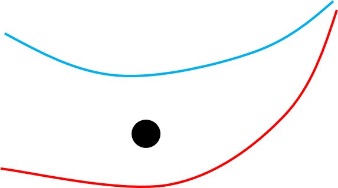


**Figure S8.** **Split and non-split pairs.** (**a**) and (**b**) show examples of non-split bat pairs. (**c**) shows example of a split bat pair. The red and blue curves indicate the trajectories of the leader and the follower. The black dot indicates the position of the pole.


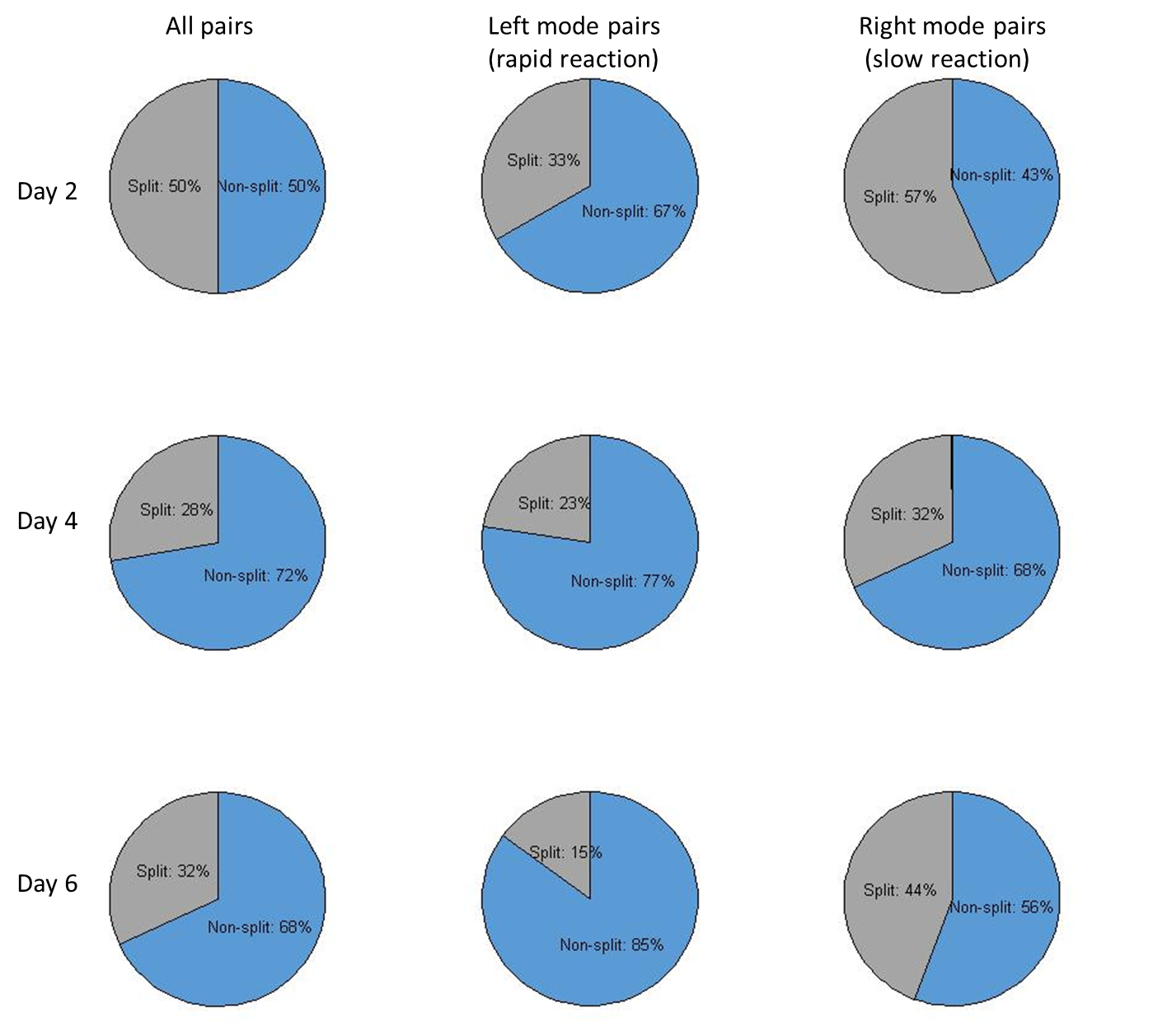


**Figure S9.** **Split and non-split pairs in each mode.** The percentages of split and non-split pairs for all bat pairs, those in the left mode (corresponding to rapid alignment between the pair) and those in the right mode (corresponding to slow alignment between the pair).

**Figure S10.** **Scatter plot of the heading delay and the average distance between leader and follower for leader-follower bat pairs on Day 2.**

**References**

[1] Theriault, D. H. *et al.* A protocol and calibration method for accurate multi-camera ﬁeld videography. *J. Exp. Biol.* **217**, 1843–1848 (2014).
